# A multi-pass differential photoacoustic spectrometer at 1064 nm for ambient aerosol absorption

**DOI:** 10.1016/j.pacs.2025.100769

**Published:** 2025-09-10

**Authors:** Jie Chen, Xianmei Qian, Wenyue Zhu, Qiang Liu, Jianjie Zheng, Tao Yang, Tengfei Yang

**Affiliations:** a State Key Laboratory of Laser Interaction with Matter, Anhui Institute of Optics and Fine Mechanics, HFIPS, Chinese Academy of Sciences, Hefei 230031, China; b University of Science and Technology of China, Hefei 230026, China

**Keywords:** Photoacoustic spectroscopy, Multi-pass differential photoacoustic spectrometer, Atmospheric aerosol, Absorption coefficient

## Abstract

Accurate atmospheric aerosol absorption measurements are critical for advancing our understanding of global climate effects and reginal meteorological processes. In this paper, a multi-pass differential photoacoustic spectrometer (MP-DPAS) worked at 1064 nm, was developed for the in-situ measurement of atmospheric aerosol absorption coefficients (Abs). By employing the multi-pass configuration, 22 reflections of the incident laser were achieved, thereby the photoacoustic signal was enhanced by a factor of ten. Meanwhile, the differential configuration not only suppress background noise but also amplifies the signal by a factor of two. Consequently, the MP-DPAS achieved a minimum detection limit of 0.05 Mm^−1^ within an integration time of 110 s and a precision of 1.4 Mm^−1^. The accuracy of the MP-DPAS was validated by comparing the measured Abs with the calculated Abs of Mie scattering theory and the variation of particle size distribution measured by SMPS (Scanning Mobility Particle Sizer).

## 1 Introduction

Aerosol light absorption stands as a key parameter for accurately assessing Earth's atmospheric radiative effects and climate impacts [1], [2], [3]. Photoacoustic spectroscopy is the most reliable and accurate measurement technique for determining the light absorption coefficient of atmospheric aerosols [4]. Of particular significance are the multi-wavelength photoacoustic instruments that have emerged in the past decade, which are not only capable of determining the optical absorption coefficient or the mass concentration of BC aerosols, but are also suitable for air quality monitoring and the identification of emission sources [5], [6], [7], [8]. Given its high sensitivity and capability for in-situ measurement, photoacoustic spectroscopy has been widely employed in aerosol and gas detection [9], [10], [11], [12], [13], [14], [15], [16], [17], [18], [19], [20]. As one of the most sensitive absorption spectroscopy techniques, photoacoustic spectroscopy is limited by environmental acoustic noise, airflow noise and coherent noise introduced by the optical system, which restricts the signal-to-noise ratio and detection limit of the photoacoustic spectrometer (PAS) [21], [22], [23], [24]. To enhance the photoacoustic signal and eliminate background noise, differential photoacoustic spectroscopy has been gradually introduced into PAS. The differential photoacoustic cell (DPAC) was initially introduced by Busse and Herber as a differential photoacoustic detector to enhance the signal and reduce noise [25].

At present, there are three types of differential photoacoustic spectroscopy sensors: (1) DPAC structure, (2) dual optical path systems, and (3) differential mode excitation photoacoustic (DME-PA) technique. The most prevalent differential photoacoustic spectrometer (DPAS) configurations were realized by employing two symmetrical acoustic resonators as DPAC [26], [27]. Acoustic resonators perform key tasks in gas sensing, including gas collection, suppression of environmental acoustic noise, and enhancement of photoacoustic signal [28]. Ma et al. demonstrated highly sensitive detection of ethylene (C_2_H_2_) by designing a differential Helmholtz photoacoustic cell (DHPAC) and concurrently integrating Erbium-doped fiber amplifier (EDFA) with multi-pass cell [29]. Fu et al. designed a micro-scale DHPAC and simulated the acoustic behavior as well as the gas flow noise within the Helmholtz photoacoustic cell (PAC) [30], [31]. Based on this design, the resonant cavity volume was merely 0.5 ml, and a minimum detection limit of 17.9 ppb for methane (CH_4_) was successfully achieved. DPAC structure, which utilize two microphones with subtracted output signals to eliminate common-mode noise, are capable of reducing environmental noise, flow noise, and certain cell wall interference.

Dual-path optical system introduces two light beams into the sample cell and the reference cell respectively, aiming to suppress the adverse effects of coherent noise, thereby revealing the hidden weak absorption. The structure of using two completely identical and separate absorption cavity as PAC is a commonly configuration in dual-path DPAS. Kapitanov et al. used a near infrared semiconductor laser as the light source and directed the light into two novel ring type resonators to measure the absorption spectrum of CH_4_ in 6080–6180 cm^−1^ region [32]. The system was capable of measuring weak absorption coefficients as low as 1.4 × 10^−7^ cm^−1^ Hz^−1/2^ while the laser power was 3 mW. Using a similar setup, Zhu et al. demonstrated aerosol absorption measurements at 1342 nm with vapor absorption interference, and Cao et al. successfully measured aerosol absorption and nitrogen dioxide (NO_2_) concentration simultaneous based a 443 nm diode laser [33], [34].

DME-PAS is based on the frequency division multiplexing (FDM) method, which exciting two longitudinal modes of a single PAC. The gas light absorption signals can be extracted by calculating the amplitude ratio of the two demodulated acoustic signals. With this method, Karhu et al. presented a highly sensitive DME-PAS NO_2_ sensor utilizing a commercially available white light diode [35]. The light source was split into two beams through a dichroic filter, and the separated beams were frequency-multiplexed and combined before sending into the same photoacoustic cell. Photoacoustic signals were measured while simultaneously calculating NO₂ concentration.

To the best of our knowledge, while there have been numerous reports on DPAS for measuring aerosol absorption and trace gas concentrations, few DPAS instruments have been successfully applied to atmospheric aerosol absorption measurements in the near infrared band due to insufficient sensitivity [36], [37], [38], [39], [40]. In this paper, a multi-pass differential photoacoustic cell (MP-DPAC) was developed and the key performances of that were simulated using the finite element method (FEM). Based on the MP-DPAC design, the MP-DPAS demonstrated aerosol absorption measurements at 1064 nm. The accuracy of the MP-DPAS was validated by comparing measured Abs with the calculated values of Mie scattering theory and the variation of particulate matter measured by SMPS (TSI, 3936).

## 2 Development of the MP-DPAS

### 2.1 PA theory

The photoacoustic signal (S_PA_) [V] detects by microphone, directly related to the power of the incident light *P* [W] as described below [41]:

(1) $$S_{PA}=P\times M\times C_{\mathrm{cell}}\times \alpha \times c$$

The sensitivity of microphone is indicated by *M* [V/Pa], while the cell constant is expressed as *C*_cell_ [Pa/W Mm^−1^]. The parameters *α* [Mm^−1^/ppbv] and *c* [ppbv] represent the mass-specific absorption cross-section and the concentration of the absorbing material, respectively. According to Eq. (1), M, *P* and *α* are the primary determinants of the photoacoustic signal amplitude.

### 2.2 Design and theoretical analysis of the MP-DPAC

**Fig. 1:**
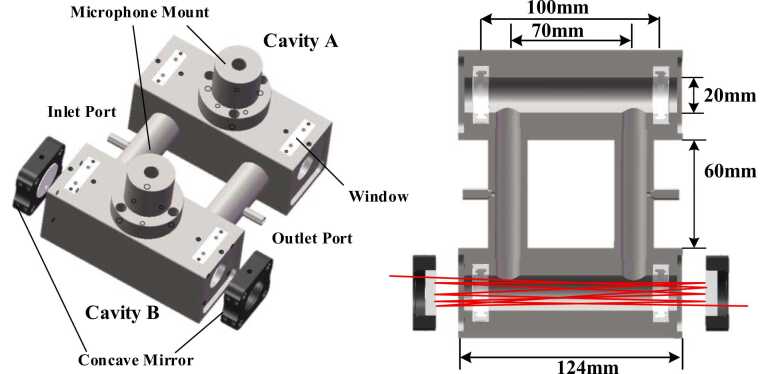
Structure of the MP-DPAC.

The designed MP-DPAC structure is shown in Fig. 1. It comprises a DPAC integrated with two silver-coated concave mirrors. The DPAC contains two parallel cylindrical cavities (20 mm inner diameter × 100 mm length) of identical dimensions connected by two tubes (12 mm inner diameter × 60 mm length). Each cavity has a central port for microphone mounting, while the tubing incorporates gas inlet/outlet ports for sample exchange. The entire assembly was fabricated from stainless steel. The concave mirrors (2 in. outer diameter, 200 mm focal length) were installed at both ends of cavity B. This configuration forms the MP-DPAC system upon laser irradiation of cavity B, enabling significant amplification of the photoacoustic signal.

**Fig. 2:**
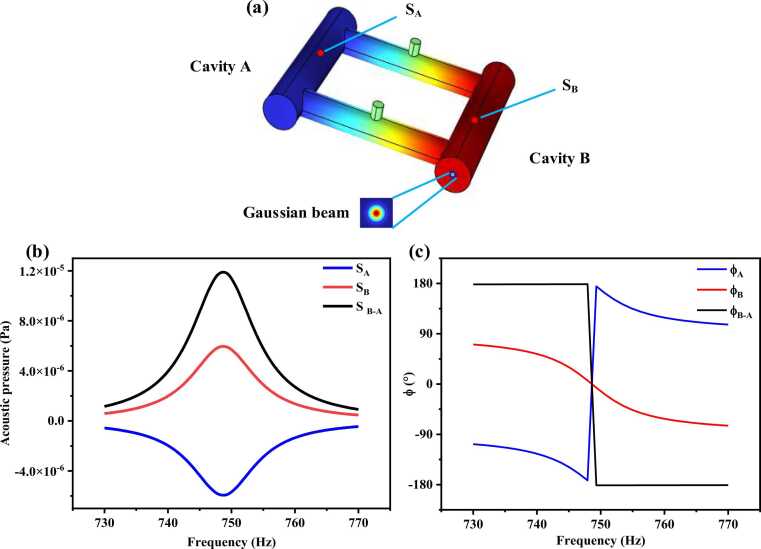
(a) Finite element model of DPAC (b, c) simulated frequency dependence of acoustic pressure amplitude and phase by FEM.

FEM analysis was conducted using the thermo-viscous acoustics module in the COMSOL Multiphysics® v6.1 to simulate the three-dimensional acoustic pressure distribution within the DPAC and the result are given in Fig. 2(a). The incident light is Gaussian beam and the simulation details are consistent with those in prior literature [31]. The frequency dependence of the amplitude (S_A_, S_B_) and phase (φ_A_, φ_B_) of the acoustic pressure at the midpoints of cavity A and B and their difference (S_B-A_, φ_B-A_) are shown in Fig. 2(b, c). The simulation results indicate that the midpoint of cavity B corresponds to an antinode of the acoustic wave, while the midpoint of cavity A corresponds to a node, with a phase difference of 180°. The differential configuration amplifies the signal by a factor of two and suppresses common-mode noise of the both cavities. Additionally, the fundamental resonant frequency of the DPAC is confirmed to be 749 Hz.

**Fig. 3:**
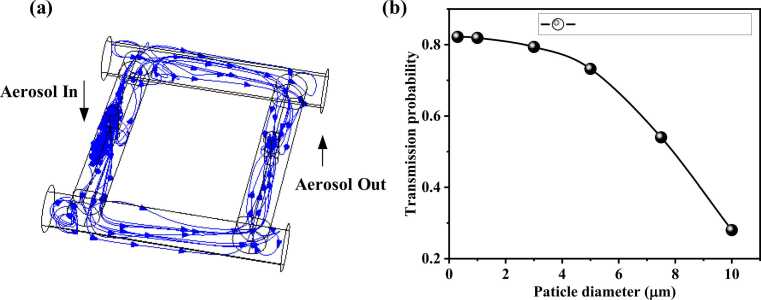
(a) Aerosol trajectories and (b) transmission probability in the DPAC.

One of the most important requirements in aerosol sampling is to ensure that aerosol particles can effectively pass through the measurement device. Based on COMSOL, we also conducted a simulation study on the loss of particles with diameters ranging from 0.3 μm to 10 μm in the DPAC. As shown in Fig. 3(a), it presents the transmission trajectory of particles in the DPAC, and Fig. 3(b) shows the transmission probability of particles with different diameters. It can be seen from Fig. 3(b) that the measurement device has a more significant loss for larger particle sizes. When the particle diameter is higher than 10 μm, the transmission probability of particles is lower than 0.28, and they almost do not pass through. Therefore, it can be approximately considered that the cut-off diameter of aerosol particles in the DPAC is 10 μm.

**Fig. 4:**
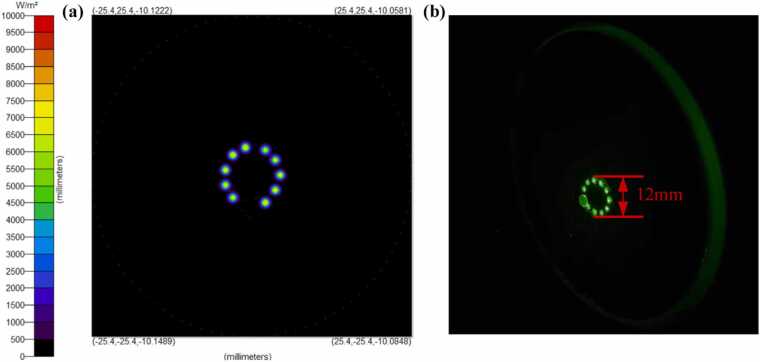
(a) Simulated and (b) measured photographs of the beam patterns on the mirror.

Fig. 4(a, b) present the simulated and experimental spot distributions of the multi-pass configuration, respectively. Accounting for unobstructed beam propagation through the absorption cavity, 22 reflections were achieved (corresponding to an optical path length of 5.06 m). As measured in Fig. 4(b), the beam diameter at the mirror surface is approximately 12 mm.

In the case of MP-DPAC, the effective laser power within the PAC can be calculated using the formula provided below [42]:

(2) $$P_{\text{eff}}=\overset{\underset{n}{k=0}}{\sum }PR^{n}$$

where *P* is the output power of the laser (940 mW), *R* stands for the reflectivity of the concave mirror (approximately 92.5 % in this study), and *n* indicates the reflection number of the laser. *P*_eff_ denotes the integrated optical power achieved through the multi-pass configuration. This configuration was engineered to achieve a tenfold increase in optical power. According to Eq. (1), this significantly increases the photoacoustic signal amplitude.

With the aforementioned design, the developed MP-DPAC significantly boosts the optical power through multi-pass amplification. This enhancement theoretically increases the photoacoustic signal amplitude by a factor of ten. Furthermore, the differential detection technique doubles the photoacoustic signal while simultaneously suppressing noise. This innovative design promises to substantially enhance the detection sensitivity of photoacoustic spectroscopy. Here, it is noteworthy that the photoacoustic signal intensity could, in principle, be further enhanced by increasing the number of laser passes within the resonator. However, due to the finite reflectivity of the mirrors, the total integrated optical energy inside the cavity does not increase significantly beyond 22 reflections, leading to smaller increments in photoacoustic signal. Moreover, higher reflection number would necessitate a larger resonator diameter to decrease thermal wave generation from beam impacts on the resonator walls. This increase in resonator size would reduce the energy density of the resonator ultimately attenuating the photoacoustic signal strength. Thus, for the present resonator geometry, an optimal balance was achieved at 22 reflections, maximizing signal enhancement while preserving energy density.

### 2.3 MP-DPAS setup

**Fig. 5:**
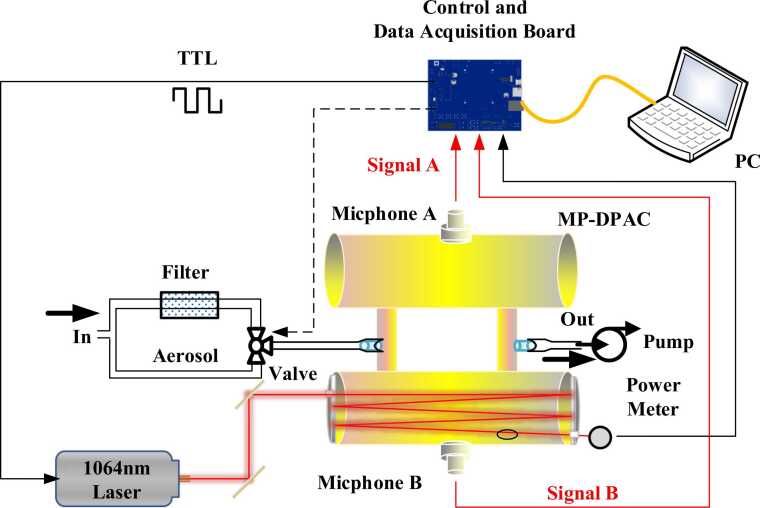
Setup of 1064 nm MP-DPAS for aerosol absorption measurement.

Fig. 5 depicts the MP-DPAS for aerosol absorption measurement, which includes three functional modules: (1) Optical module: 1064 nm laser (CNI, MIL-H-1064-T), MP-DPAC, and optical power meter (OPHIR, NOVA II); (2) Airflow control module: tubing, particulate filter (Parker, 9922–05-BQ), solenoid valve, and pump (Kamoer, KVP04–1.1–12); (3) Electronics module: microphones (BSWA, MA221), home-made control/data acquisition board, and PC. The control/data acquisition board provides TTL signal generation, photoacoustic signal acquisition/differential processing, lock-in amplification, laser power monitoring, and automated gas path switching.

TTL-modulated laser radiation illuminates the MP-DPAC, inducing photoacoustic signals through periodic aerosol heating. Dual microphones in differential configuration convert acoustic waves into electrical signals, specifically suppressing common-mode noise while enhancing the axisymmetric photoacoustic response. This differential signal undergoes lock-in amplification synchronized to the laser modulation frequency to isolate the weak photoacoustic component. Sample airflow was regulated at 500 ml/min, with automated background subtraction implemented via a solenoid valve that periodically switches (every 30 min) between aerosol-laden sample air and particle-filtered reference air. Laser power was continuously monitored by the power meter for signal normalization, while a custom LabVIEW program executes all control, data acquisition, signal processing, and absorption coefficient calculation.

## 3 Results and discussion

### 3.1 Resonant frequency and quality factor

**Fig. 6:**
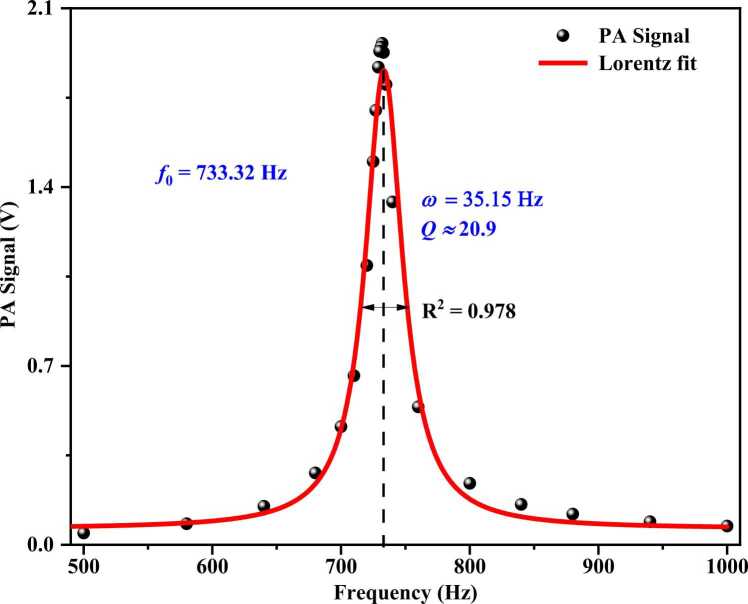
Measured frequency response of MP-DPAC.

Modulation frequency serves as a critical determinant of photoacoustic signal amplitude, with system optimization achieved through matching of the modulation frequency to the MP-DPAC resonant frequency (*f*_0_). The *f*_0_ was acquired by frequency scanning with high concentration of aerosols. As shown in Fig. 6, the frequency response of the PAC exhibits a Lorentzian profile, with a measured resonant frequency of 733.32 Hz and quality factor (Q, Q = *f*_0_÷ω, ω, full width at half maximum) of 20.9. The measured resonant frequency shows only 2 % deviation from the simulation results, demonstrating good agreement between the experimental and simulated data. Furthermore, all subsequent experiments were conducted at this resonant frequency.

### 3.2 Calibration of the MP-DPAS

Prior to aerosol absorption measurement, the MP-DPAS was calibrated with the absorption of NO_2_ at 532 nm as mentioned in [33]. The concentration of NO_2_ was monitored synchronously by NO_x_ analyzer (Thermo, 42i), and the effective absorption cross-section was calculated with the emitting spectrum of the 532 nm laser (CNI, MGL-U-532–1W) and the absorption cross-section from the HITRAN database [43].

**Fig. 7:**
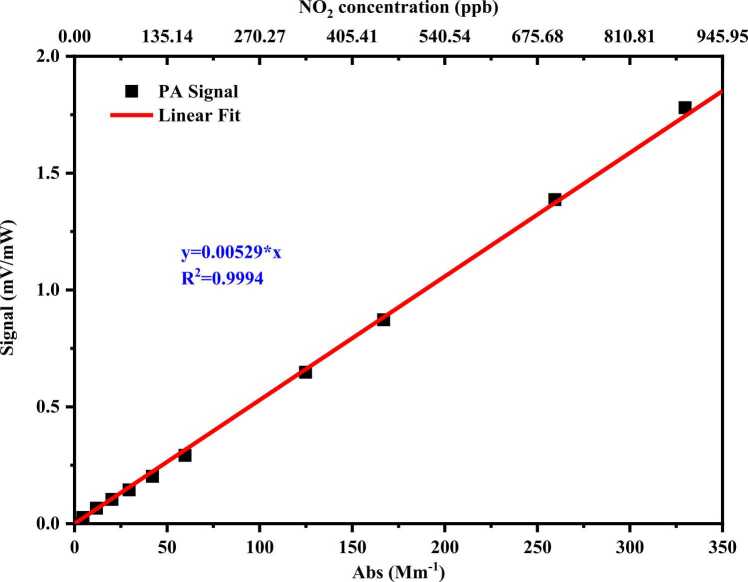
Calibration results of MP-DPAS.

The MP-DPAS was calibrated using certified NO₂ reference gases spanning concentrations of 0–1 ppm. The photoacoustic signal was linearly fitted against the corresponding NO_2_ Abs at each concentration, yielding the system calibration curve shown in Fig. 7. The slope of this curve represents the system's calibration factor. As depicted in Fig. 7, the linear fit exhibits an excellent correlation coefficient (R²) of 0.9994, with a determined calibration factor of 5.29 × 10^−3^ mV·Mm^−1^·mW^−1^. In all subsequent measurements, aerosol absorption coefficients were determined via inversion by this calibration factor.

### 3.3 Performance evaluation of MP-DPAS

To validate the enhancement effect of the multi-pass configuration on MP-DPAS, we conducted experiments comparing the signal of three structures: the single-channel PAC, DPAC, and MP-DPAC. A fixed-concentration nigrosine aerosol was introduced into the photoacoustic cell, and photoacoustic signal were measured under identical conditions.

**Fig. 8:**
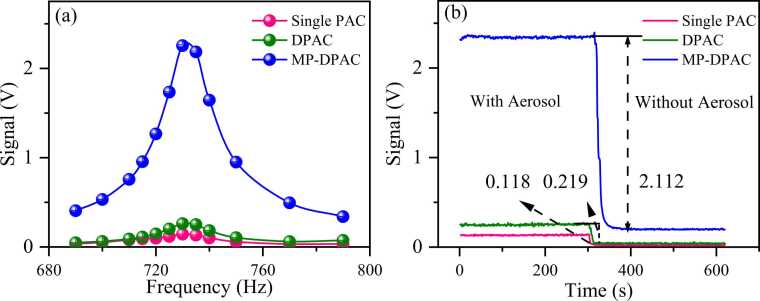
**(a)** Frequency response of photoacoustic signals based on different cavity structures (b) absorption signals of aerosols with a fixed concentration for single PAC, DPAC and MP-DPAC, respectively.

Fig. 8(a) compares the frequency-scanned signals across the three configurations, while Fig. 8(b) shows the photoacoustic amplitudes at the resonant frequency (733 Hz). The absorption signal was calculated based on the change in photoacoustic signal amplitude before and after aerosol filtration. As shown in Fig. 8(b), the MP-DPAC signal was 9.6 times stronger than DPAC and 17.9 times higher than the single-channel cell, consistent with the expected ∼10 times enhancement over conventional DPAS.

**Fig. 9:**
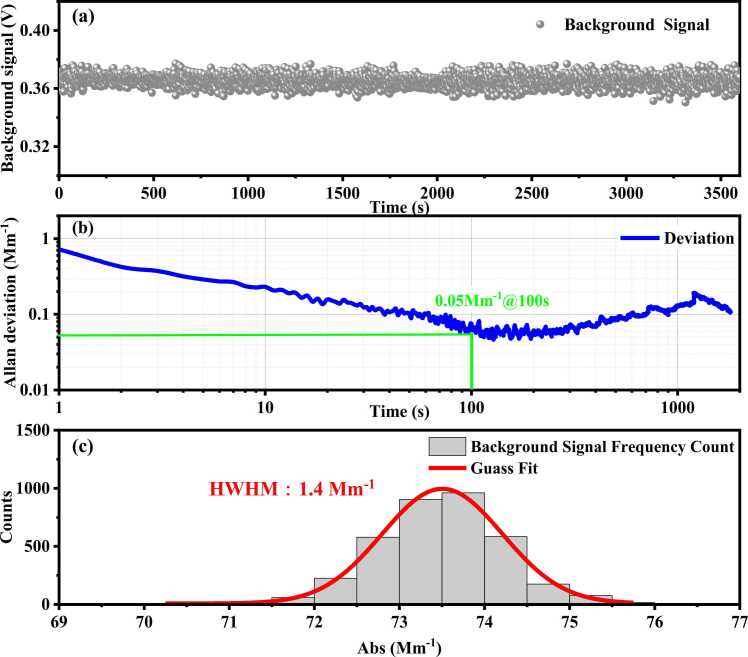
Performance analysis of the MP-DPAS, (a) time series of the background signal (b) Allan deviation from background signal (c) histogram from background signal.

The MP-DPAS performance was further characterized through analysis of the background signal time series under aerosol-free conditions. Furthermore, the system exhibited a data resolution of 1 s. Fig. 9(a) presents the 1 h background signal time series acquired by the system. Fig. 9(b) displays the sensitivity results derived from Allan deviation analysis of this background signal. The MP-DPAS exhibits detection limits ranging from approximately 0.73 Mm^−1^ at 1 s integration time to 0.05 Mm^−1^ with integration time extended to 110 s. Fig. 9(c) presents the frequency distribution from the background signal. This histogram characterizes the MP-DPAS measurement precision, where the half width at half maximum (HWHM) of the Gaussian fit yields a precision of 1.4 Mm^−1^ at 1 s integration time.

To evaluate the detection sensitivity of the photoacoustic system in this study, the detection limits of the MP-DPAS and other reported PAS sensors in the visible and near-infrared bands are compared in Table 1. It can be seen that the detection limit of MP-DPAS is superior to or essentially consistent with the reported results. Particularly in the near infrared band, the MP-DPAS can be employed to further enhance the detection limit of PAS and to effectively obtain the aerosol absorption coefficient.

**Table1:** Performance comparison of photoacoustic aerosol absorption measurement devices.

Spectrometer configuration	Wavelength (Power)	Detection sensitivity （Integration time）	Ref
Non-resonant PAC and cantilever microphone	532 nm (1 W)	0.013 Mm^−1^ (10 s)	Karhu et al. [44]
DPAC and microphone	1342 nm	0.3 Mm^−1^ (120 s)	Liu et al. [33]
DPAC with MEMS microphone	473 nm (150 mW) 532 nm (300 mW) 671 nm (200 mW)	0.9 Mm^−1^ 0.22 Mm^−1^ (100 s) 0.2 Mm^−1^	Yu et al. [45]
Symmetric multi-resonator PAC with microphone	404 nm (170 mW) 637 nm (360 mW) 805 nm (660 mW)	0.78 Mm^−1^ 0.31 Mm^−1^ (200 s) 0.2 Mm^−1^	Cao et al. [46]
Dual-T-type PAC with microphone	532 nm	0.95 Mm^−1^	Li et al. [47]
Single PAC and microphone	406 nm (100 mW) 532 nm (80 mW) 662 nm (100 mW) 785 nm (100 mW)	0.71 Mm^−1^ 0.62 Mm^−1^ (120 s) 0.61 Mm^−1^ 0.75 Mm^−1^	Fischer and Geoffrey [37]
Multi-PACs and microphone	266 nm (5 mW) 355 nm (12 mW) 532 nm (100 mW) 1064 nm (450 mW)	35.5 Mm^−1^ 23.5 Mm^−1^ 5.2 Mm^−1^ 0.2 Mm^−1^	Ajtai et al. [39]
MP-DPAC and microphones	1064 nm (940 mW)	0.05 Mm^−1^ (110 s)	This work 2025

### 3.4 Comparison of measured and calculated absorption coefficients

Mie scattering theory was employed to compare the theoretical absorption parameters with the experimental results in this study. Since the refractive index of nigrosine aerosol at 1064 nm was not clearly recorded in previous reports, in order to accurately calculate the absorption efficiency of the aerosol, this study conducted experiments using the absorption of nigrosine aerosol at 532 nm and compared the experimental results with the theoretical calculation. It is worth noting that the sole difference from the aforementioned 1064 nm MP-DPAS lies in the replacement of the light source with a 532 nm laser. This alteration in the light source has no impact on the theoretical validation results.

**Fig. 10:**
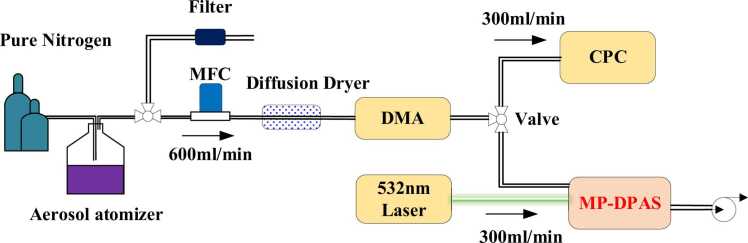
Experimental setup for production and measurement of monodisperse nigrosine aerosol.

Nigrosine aerosol has been extensively utilized to validate the accuracy of instrument measurements in cavity ring-down spectroscopy (CRDS), broadband cavity enhancement spectroscopy (BCEAS), and photoacoustic spectroscopy [48], [49], [50], [51]. The aerosol particle size classification system is illustrated in Fig. 10. The detailed procedure has been extensively employed in prior studies and thus will not be further elaborated upon in this study [52], [53]. Different from previous studies, a range of diameters (nominally 50 nm, 250 nm, 350 nm, 450 nm, 550 nm, 650 nm, 750 nm, 850 nm and 1000 nm) were obtained for this analysis.

Herein, the Abs can be calculated using the following equation:

(3) $$Abs\left(\lambda \right)=\int Q_{Abs}\left(\lambda ,D_{p},m\right)\frac{\pi D_{p}^{2}}{4}N\left(D_{p}\right)d\;\log\;D_{p}$$

*Q*_Abs_ denotes the absorption efficiency calculated with the Mie code of Barber and Hill. For further details on the other parameters in the Eq. (3) and their descriptions, please refer to [54]. The complex refraction (*m*) of a nigrosine aerosols at 532 nm (*m*=1.7 +0.31 i) was referred to the previous literature [53]. For monodisperse aerosols, the upper formula can be simplified to the following formula:

(4) $$Abs\left(\lambda \right)=N\left(D_{p}\right)\cdot \delta_{Abs}\left(\lambda ,D_{p},m\right)$$

(5) $$\delta_{Abs}\left(\lambda ,D_{p},m\right)=Q_{Abs}\left(\lambda ,D_{p},m\right)\cdot \delta \left(D_{p}\right)$$

where *δ*_Abs_ designates the absorption cross-section calculable by the Mie theory, and *δ* refers to the geometric cross-sectional area of the particle.

**Fig. 11:**
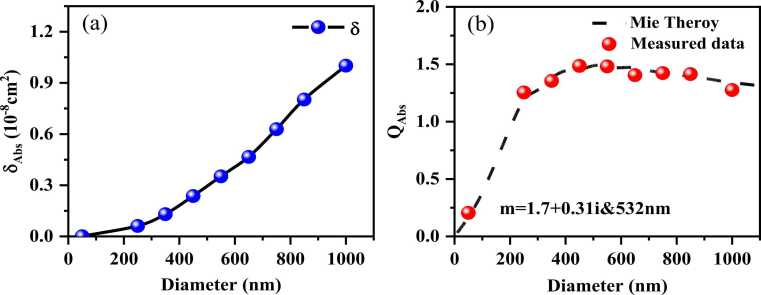
Comparison of experimental measurement with theoretical calculation of nigrosine aerosol at 532 nm, (a) the measurement values of *δ*_Abs_ (b) *Q*_Abs_ of nigrosine aerosol calculated based on the Mie theory and experimental results.

Fig. 11(a) shows the measurement values of *δ*_Abs_ of aerosol particles with different sizes. Assuming spherical particles, the Mie theory was hereby adopted to determine *Q*_Abs_. In Fig. 11(b), the black dashed line indicates the calculation results of the Mie theory, and the red dots represent the experimental measurement results, showing excellent consistency.

## 4 Atmosphere measurements

The light absorption of atmospheric aerosols at 1064 nm, measured using the MP-DPAS, was conducted at HFIPS (31.893 N, 117.171 E) at a sampling location approximately 5 m above the ground. The sampling location in the suburban of Hefei city is influenced by rural activities, urban vehicle exhaust, and industrial emissions. During measurement, aerosol concentration and particle size distribution were determined using an SMPS and compared with the aerosol absorption coefficient obtained from the MP-DPAS. When measuring the absorption characteristics of aerosols in a high-humidity environment, water vapor evaporation can obscure the photoacoustic signal. Therefore, a diffusion dryer at the spectrometer inlet first removes water vapor from sampled air. The MP-DPAS sampling flow was set to 500 ml/min, and the data resolution was 1 s. Sampling time of the SMPS was set to 180 s per cycle, with a particle diameter measurement range of 13.8–697.8 nm.

**Fig. 12:**
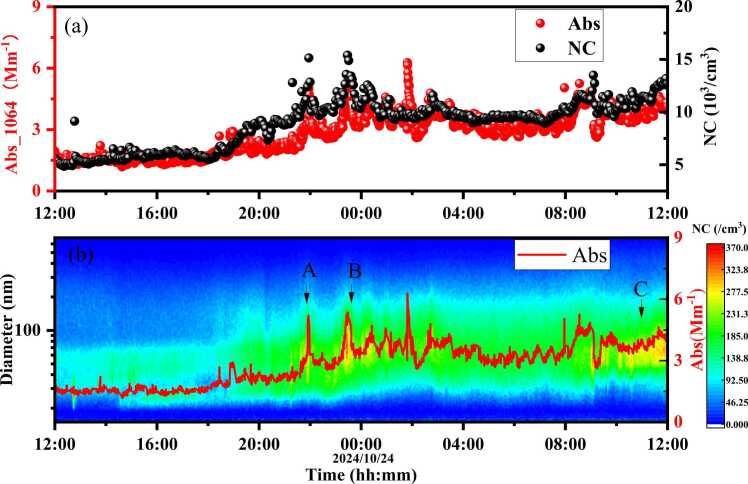
Time series measurements of atmospheric aerosols, (a) comparison of Abs at 1064 nm measured by the MP-DPAS and particle number concentration (NC) measured by SMPS (b) time variation of Abs at 1064 nm and the aerosol particle size distribution.

The measurement results of atmospheric aerosol light absorption and size distribution are presented in Fig. 12. The red points in Fig. 12(a) represent Abs measured by the MP-DPAS, and the black points indicate aerosol concentration, showing good consistency. Fig. 12(b) shows the relationship between the aerosol Abs and the aerosol particle size spectrum over time. As shown in Fig. 12(b), small particle bursts at A, B, and C lead to short-term increases in aerosol absorption coefficient, with MP-DPAS better reflecting aerosol evolution. It is worth noting that, in this study, while the absorption coefficient measured by the MP-DPAS demonstrates good consistency with the particle size distribution obtained from the SMPS, subtle discrepancies still exist. These differences primarily arise from the distinct measurement principles of the two techniques: MP-DPAS quantifies aerosol absorption coefficients, while SMPS measures particle size distributions. The absorption coefficient is influenced by a combination of factors, including particle size, chemical composition, and meteorological conditions. Although SMPS data can partially reflect variations in absorption properties, it cannot fully capture all contributing factors, leading to the observed discrepancies. In subsequent research, we aim to further refine the MP-DPAS to enhance its measurement accuracy and sensitivity for aerosol absorption coefficients.

## 5 Conclusion

In this study, we present a high-sensitivity MP-DPAS operating at 1064 nm, specifically designed for in situ measurements of atmospheric aerosol absorption. The DAPC of MP-DPAS combined with multi-pass amplification achieves a tenfold increase in effective laser power and twofold signal enhancement while suppressing common-mode acoustic noise. Finite element simulations validated the resonator frequency at 733.32 Hz, enabling system optimization. The instrument attains a minimum detection limit of 0.05 Mm^−1^ at 110 s integration time and 1.4 Mm^−1^ precision at 1 s, representing one of the most sensitive systems reported for near-infrared photoacoustic aerosol detection. Validation with monodisperse nigrosine aerosols showed agreement with Mie theory predictions. During 24-hour ambient monitoring, aerosol absorption dynamics exhibited strong correlation with SMPS-derived particle size distribution evolution, capturing atmospheric transformation processes. The aerosol photoacoustic spectrometer developed in this study addresses the limitations of conventional PAS, such as susceptibility to multiple noise interferences and insufficient sensitivity in aerosol measurements. Furthermore, by analyzing aerosol absorption at 1064 nm, this work fills a critical gap in the understanding of aerosol absorption characteristics in the near-infrared region, where prior research has been scarce. In this manner, this study establishes the MP-DPAS as a robust tool for quantifying near-infrared aerosol absorption coefficients, providing a critical technical foundation for investigating atmospheric aerosol transformation mechanisms.

## CRediT authorship contribution statement

**Tengfei Yang:** Validation. **Tao Yang:** Software. **Qiang Liu:** Methodology, Investigation, Formal analysis, Data curation. **Jianjie Zheng:** Formal analysis. **chen jie:** Writing – review & editing, Writing – original draft, Funding acquisition, Formal analysis, Data curation. **Qian Xian mei:** Investigation, Formal analysis. **Wenyue Zhu:** Investigation.

## Declaration of Competing Interest

The authors declare that they have no known competing financial interests or personal relationships in this paper.

## Data Availability

Data will be made available on request.
